# Direct Measurement of the Visible to UV Photodissociation Processes for the PhotoCORM TryptoCORM†

**DOI:** 10.1002/chem.202001077

**Published:** 2020-07-24

**Authors:** Rosaria Cercola, Kaitlyn C. Fischer, Summer L. Sherman, Etienne Garand, Natalie G. K. Wong, L. Anders Hammerback, Jason M. Lynam, Ian J. S. Fairlamb, Caroline E. H. Dessent

**Affiliations:** ^1^ Department of Chemistry University of York Heslington York YO10 5DD UK; ^2^ Department of Chemistry University of Wisconsin-Madison Madison WI 53706 USA

**Keywords:** CORM, photoCORM, photodissociation, prodrugs, transition-metal carbonyls

## Abstract

PhotoCORMs are light‐triggered compounds that release CO for medical applications. Here, we apply laser spectroscopy in the gas phase to TryptoCORM, a known photoCORM that has been shown to destroy *Escherichia coli* upon visible‐light activation. Our experiments allow us to map TryptoCORM's photochemistry across a wide wavelength range by using novel laser‐interfaced mass spectrometry (LIMS). LIMS provides the intrinsic absorption spectrum of the photoCORM along with the production spectra of all of its ionic photoproducts for the first time. Importantly, the photoproduct spectra directly reveal the optimum wavelengths for maximizing CO ejection, and the extent to which CO ejection is compromised at redder wavelengths. A series of comparative studies were performed on TryptoCORM‐CH_3_CN which exists in dynamic equilibrium with TryptoCORM in solution. Our measurements allow us to conclude that the presence of the labile CH_3_CN facilitates CO release over a wider wavelength range. This work demonstrates the potential of LIMS as a new methodology for assessing active agent release (e.g. CO, NO, H_2_S) from light‐activated prodrugs.

## Graphical abstract

**CO release**: The application of a new technique, laser‐interfaced mass spectrometry, for characterizing the ability of a PhotoCORM (CORM=carbon monoxide releasing molecule) to release CO as a function of excitation wavelength is demonstrated. This approach also reveals the identity of the photoproducts, and has potential for application to a wide range of light‐activated prodrugs.

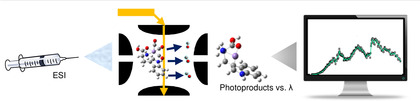

## Introduction

While the toxicity of carbon monoxide is well known, its biological significance and therapeutic properties have been increasingly recognized over recent years.^1^, ^2^ At low concentrations, CO is a powerful anti‐inflammatory and organ transplantation agent that also has significant potential as a chemotherapy and anti‐microbial pharmaceutical.^3^, ^4^, ^5^, ^6^, ^7^, ^8^ Controlled CO delivery remains a significant challenge, however, for its practical medicinal use. One solution to delivering localized CO involves the use of carbon monoxide releasing molecules (CORMs) that exhibit CO release *only* when triggered.^1^, ^2^, ^9^, ^10^, ^11^, ^12^ Transition metal carbonyl complexes that eject CO upon light activation (photoCORMs) have emerged as the most promising delivery agents to date.^13^, ^14^, ^15^ Most photoCORMs developed so far incorporate group 7 and 8 transition metals and require UV light to trigger CO release.^16^ These wavelengths presents a significant problem for wide‐scale photoCORM application as they penetrate tissue poorly, and a major challenge in this field at the present time is therefore development of new visible‐light activated photoCORMs.^17^

The rational design of future photoCORMs could be considerably improved through obtaining a more complete understanding of their fundamental photomolecular properties.^18^ A number of groups are using this developmental approach, combining known transition metal photochemistry with density functional theory calculations to predict and interpret trends for trial compounds.^19^, ^20^, ^21^ However, progress is currently hampered by a sparsity of experimental measurements on photoCORMs that can be straightforwardly compared to results from the computational approaches. Gas‐phase experiments would offer considerable benefits in this context, since results could be directly linked to computational results without deploying the very advanced calculations that are necessary to properly account for solvent effects on the photochemistry.^22^

**Scheme 1:**
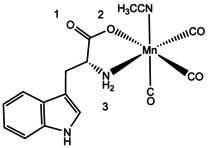
The molecular structure of TryptoCORM.^24^ The tryptophan ligand corresponds to the deprotonated form. Atom labels indicate the most likely protonation sites.

In this paper, we demonstrate a new method that has the potential to contribute significantly to progress in this area by applying UV‐VIS laser photodissociation spectroscopy to a known photoCORM, TryptoCORM (Scheme 1), to map its photochemistry at a previously unprecedented level of detail.^23^, ^24^, ^25^ This is the first time that the photochemical properties of a photoCORM have been investigated in the gas phase, thus providing crucial experimental data to evaluate recent theoretical strategies against.

Our experimental technique involves transferring the photoCORM from solution into the gas‐phase via electrospray ionization and then using novel Laser‐Interfaced Mass Spectrometry (LIMS) to measure the gaseous absorption spectrum along with all of the ionic photoproducts produced at each scanned wavelength.^26^, ^27^ Thus, we obtain a complete picture of the excited states along with the accompanying photodissociation pathways. We complement these measurements with Cryogenic Ion Vibrational Spectroscopy (CIVS),^28^ an advanced experimental technique that provides structural characterization of gaseous ions.

By directly measuring the wavelength dependence of CO ejection from TryptoCORM, we are able to demonstrate a new approach to assessing the CO releasing property of the photoCORM. This represents a significant alternative to the current principal method for determining this property, namely the myoglobin (Mb) assay.^29^ Motterlini and co‐workers were the first to report this assay, which has subsequently been refined by other research groups.^30^, ^31^ The technique measures CO release into solution indirectly through its conversion of deoxy‐Mb to Mb‐CO. The approach can be problematic, however, if CO loss from the CORM is reversible. In contrast, the new LIMS approach presented in this study provides a direct picture of the (non‐reversible) amount of CO photoejected as a function of wavelength, giving a compelling complementary approach to test Mb assays against and providing key information for selecting excitation wavelength versus amount of CO released.

TryptoCORM was the first visible‐light activated CORM to exhibit a potent effect against *Escherichia coli*.^24^ One of its key advantages as a potential therapeutic agent is that it shows low toxicity towards mammalian cells and releases bio‐benign Tryptophan on photoexcitation in aqueous solution.^24^ TryptoCORM is thought to release up to 3 CO molecules upon aqueous‐phase excitation at 400 nm (3.1 eV) dependent on conditions.^24^ However, at the lower excitation energies (465 nm, 2.66 eV) that are preferred for medical applications,^32^ fewer CO molecules are released (1.4 equivalent). The CH_3_CN group of TryptoCORM is labile,^33^, ^34^ so that the TryptoCORM‐CH_3_CN complex (i.e. the TryptoCORM molecule minus the acetonitrile ligand) is likely to exist in a dynamic equilibrium with TryptoCORM in solution, leading to questions around the relative photochemical and hence medicinal effectiveness of the two species. In this work, we are able to directly compare the photochemistry of the TryptoCORM and TryptoCORM‐CH_3_CN moieties for the first time since our mass spectrometry based technique allows us to independently isolate both molecular species prior to photoexcitation.

## Results and Discussion

### Characterization of the gas‐phase structures of electrosprayed TryptoCORM

The protonated pseudo molecular ion, [MnL(CO)_3_(CH_3_CN)]⋅H^+^, where L=deprotonated Tryptophan, appeared strongly upon electrospray of TryptoCORM (Section S1). The most intense ion observed was the protonated molecular species without the CH_3_CN ligand, that is, [MnL(CO)_3_]⋅H^+^. We note that protonation of TryptoCORM is enhanced through the electrospray process, rather than [MnL(CO)_3_(CH_3_CN)]⋅H^+^ being dominant in solution.^25^

**Figure 1:**
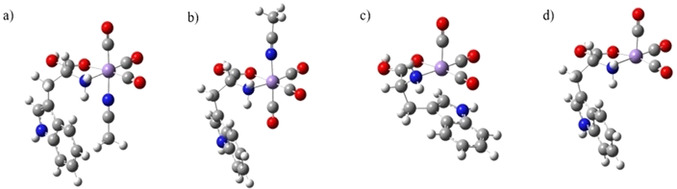
Optimized structures (PBE0‐DEF2SV) of (a) and (b) the lowest‐energy diastereoisomeric protomers of [MnL(CO)_3_(CH_3_CN)]⋅H^+^, with the (c) folded and (d) open forms of [MnL(CO)_3_]⋅H^+^.

TryptoCORM and TryptoCORM‐CH_3_CN have multiple possible protonation sites, with the most likely ones indicated on Scheme 1. Protonation on the Mn metal center is also possible for TryptoCORM‐CH_3_CN.^35^ The protonation isomer(s) formed upon electrospray can be predicted by calculating the relative energies of the various protonation site isomers or protomers.^36^, ^37^ For [MnL(CO)_3_(CH_3_CN)]⋅H^+^, the lowest‐energy gas‐phase protonation site corresponds to the O1 position (Section S2), giving rise to the structures displayed in Figures 1 a and b. This pair of diastereoisomers are predicted to be present in the ratio of 86:14 (Section S2). Protonation at other sites leads to much higher‐energy isomers that we predict are not populated.

Similarly for [MnL(CO)_3_]⋅H^+^, the lowest‐energy gas‐phase structure (Figure 1 c: a folded structure) corresponds to O1 protonation, with other protomers lying at significantly higher energies (Table S5). The O1 protomer involves the Tryptophan ligand folding to coordinate to the vacant metal center through the indole C=C bond (hapticity 2), at an interaction length (2.5 Å) that is similar to the N atom of the CH_3_CN ligand in [MnL(CO)_3_(CH_3_CN)]⋅H^+^ (2 Å). (Table S4) Coordination of the Tryptophan ligand confers significant stability on [MnL(CO)_3_]⋅H^+^, as the corresponding open structure with the indole group uncoordinated to Mn (Figure 1 d) is calculated to lie 44.6 kJ mol^−1^ above the folded structure.

**Figure 2:**
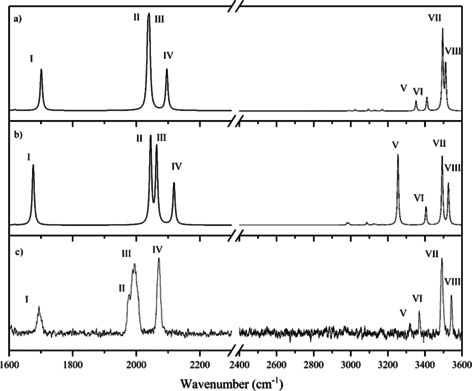
Calculated (CAM‐B3LYP‐def2SVP) vibrational spectra of the (a) folded and (b) open structures of [MnL(CO)_3_]⋅H^+^ compared to (c) the experimental IR spectrum over the ranges 1600–2300 and 2400–3600 cm^−1^. The calculated spectra are scaled with respect to wavenumber by 0.94 over the 1600–2300 cm^−1^ region, and by 0.97 over the 2400–3600 cm^−1^ region. The calculated spectral intensities for the two ranges have been scaled so that the intensities of the most intense peaks in each region are equal.

To directly test the extent of Tryptophan‐Mn coordination, cryogenic ion vibrational spectroscopy was performed on the gaseous electrosprayed ions.^28^, ^38^ Figure 2 displays the acquired IR spectrum of [MnL(CO)_3_]⋅H^+^, along with the calculated spectra of the folded (Figure 2 a) and open (Figure 2 b) structures. Table 1 lists the calculated and experimental IR frequencies.

**Table 1:** Calculated (CAM‐B3LYP/Def2SV) and experimental vibrational frequencies for the folded and open conformational isomers of [MnL(CO)3]⋅H^+^ displayed in Figure 1.

		Band	Folded ν (cm^−1^)	Open ν (cm^−1^)	Experimental ν (cm^−1^)
**CO stretch region^[a]^**	carboxylic CO	I	1701	1676	1694
	CO	II	2036	2045	1976
	CO	III	2041	2064	1994
	CO symm.	IV	2096	2118	2071
**NH/OH stretch region^[b]^**	NH_2_ symm	V	3353	3254	3323
	NH_2_ asymm	VI	3412	3404	3372
	OH	VII	3496	3492	3491
	Indole NH	VIII	3512	3525	3544

[a] Scaled by 0.94. [b] Scaled by 0.97.

Comparison of the experimental and computational spectra across the NH/OH stretch region (2400–3600 cm^−1^) confirms that the computed spectrum for the folded structure (Figure 2 a) most closely resembles the experimental spectrum (Figure 2 c), since the strong 3254 cm^−1^ vibration associated with the NH_2_ asymmetric stretch of the open structure (Figure 2 b) is absent from the experimental spectrum.

The IR spectrum of [MnL(CO)_3_]⋅H^+^ was also recorded across the CO stretching region (1600–2300 cm^−1^), with the experimental spectrum across this region displaying one CO band in the carboxylic stretch region at 1694 cm^−1^, along with three additional bands in the metal bound CO stretching region. The spectrum is consistent with previous gaseous IR spectra of metal carbonyl complexes.^39^, ^40^, ^41^, ^42^ We note that bands II and III are only partially resolved. The experimental spectrum is again consistent with the predicted spectrum for the folded structure (Figure 2 a), with the experimental bands occurring closer to the predicted vibrational frequencies for the folded structure than the open structure. IR‐IR conformer‐specific spectroscopy was performed to confirm that only a single isomer is present (Section S4).^43^

### Thermal fragmentation pathways of [MnL(CO)_3_(CH_3_CN)]⋅H^+^ and [MnL(CO)_3_]⋅H^+^

Prior to performing laser photodissociation, we investigated the thermal fragmentation pathways of [MnL(CO)_3_(CH_3_CN)]⋅H^+^ and [MnL(CO)_3_]⋅H^+^ by performing higher‐energy collisional dissociation (HCD) for energies between 0–30 % HCD. This experiment maps out the fragmentation pathways as a function of internal energy,^44^ and is therefore also of interest in the context of the known propensity of TryptoCORM to release CO upon thermal exposure, that is, to behave as a thermal‐CORM.^9^, ^23^

**Figure 3:**
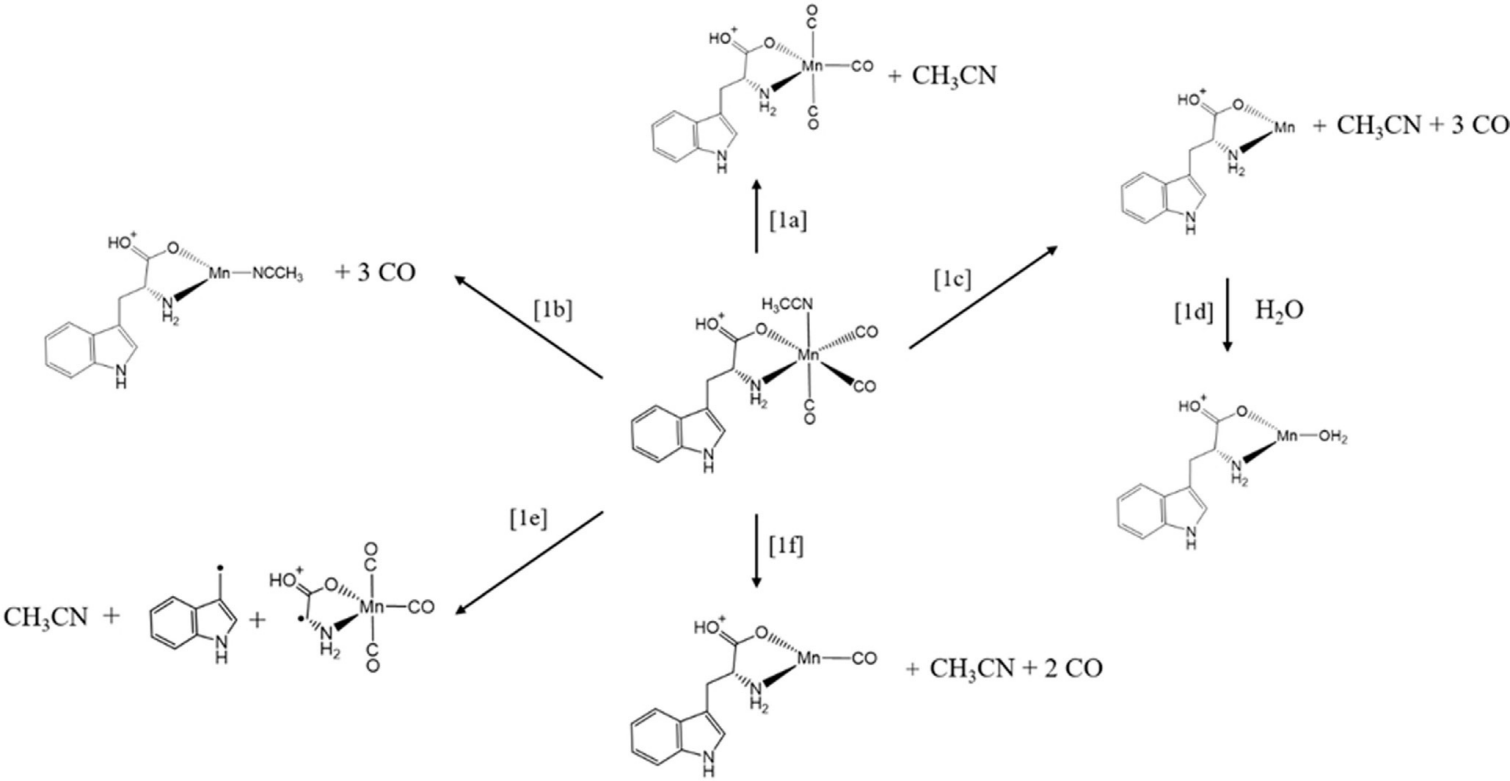
Schematic diagram to illustrate the fragmentation channels observed for [MnL(CO)_3_(CH_3_CN)]⋅H^+^ upon HCD and following photoexcitation.

The HCD data is included in Section S5, along with a detailed discussion of the various HCD channels. Figure 3 illustrates the observed [MnL(CO)_3_(CH_3_CN)]⋅H^+^ fragmentation pathways. The key observations to emerge from the HCD experiments are firstly, that the CH_3_CN ligand is very easily lost via collisional excitation, that is, the ion is metastable.^45^

Secondly, no fragmentation channel associated with loss of a single CO molecule is observed, and fragmentation with loss of two CO units is a much less intense channel than loss of three COs. It therefore seems that the barrier to loss of the second CO is low once the first CO is lost, and similarly, the barrier to 3CO loss is low once 2CO are ejected.^46^, ^47^ This situation may well be different in solution where solvation could modify these barrier heights. Finally, HCD can induce intramolecular fragmentation of Tryptophan at high collision energy. The HCD data for [MnL(CO)_3_]⋅H^+^ revealed that its fragmentation behavior closely mirrors that of [MnL(CO)_3_(CH_3_CN)]⋅H^+^, both in terms of the observed fragmentation channels and their energy dependence.

### Intrinsic (gas‐phase) electronic spectroscopy of [MnL(CO)_3_(CH_3_CN)]⋅H^+^ and [MnL(CO)_3_]⋅H^+^

**Figure 4:**
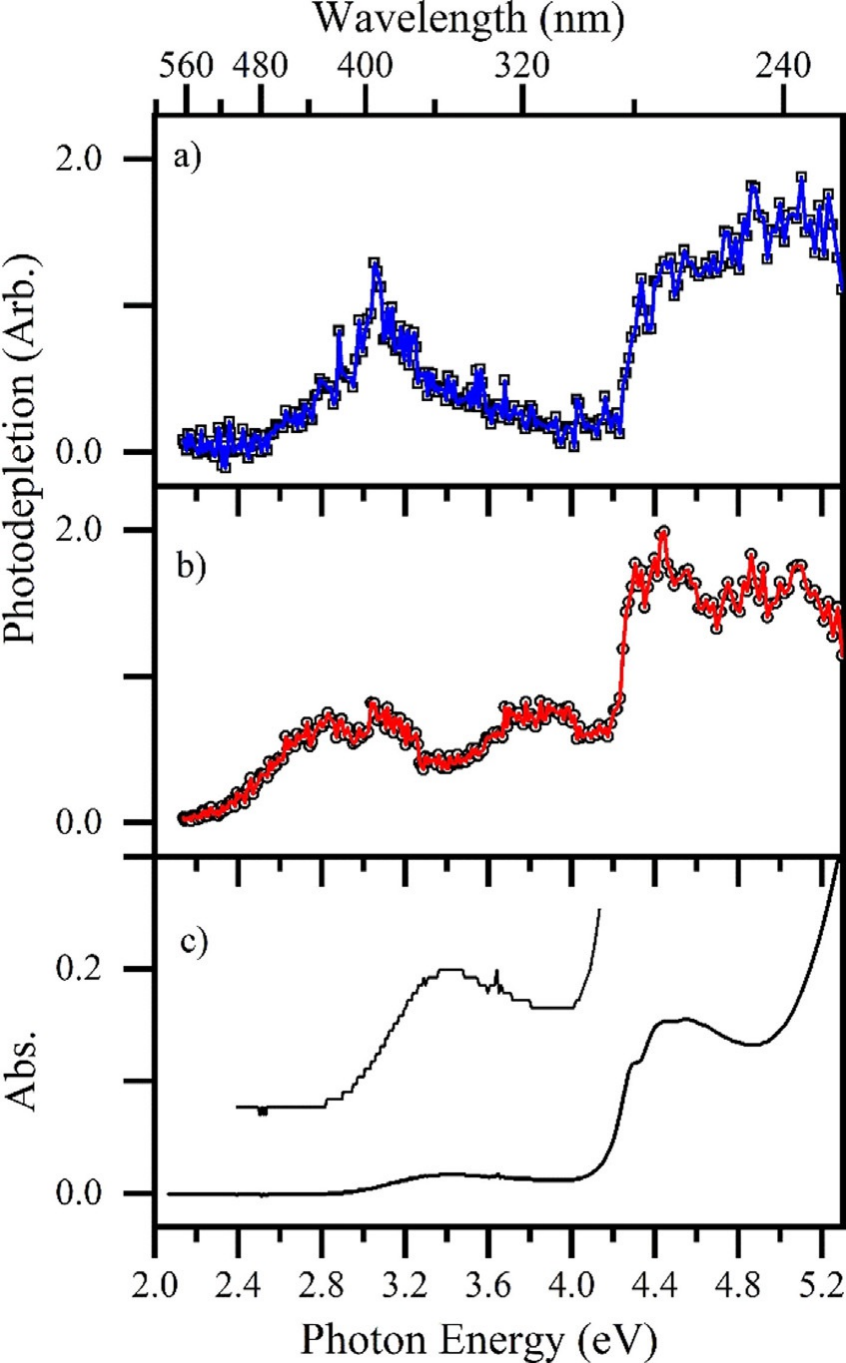
Gas‐phase absorption (photodepletion) spectra of a) [MnL(CO)_3_(CH_3_CN)]⋅H^+^ and b) [MnL(CO)_3_]⋅H^+^ (solid lines connect data points), displayed with c) the solution‐phase absorption spectrum of TryptoCORM (in H_2_O:CH_3_CN 1:1), vertically expanded for the region between 2.4–4.2 eV.

The gas‐phase absorption spectrum (recorded via photodepletion) of mass‐selected [MnL(CO)_3_(CH_3_CN)]⋅H^+^ across the 2.1–5.3 eV (234–580 nm) range is shown in Figure 4 a. Mass selection is a key feature of our experimental approach, since it means that the gas‐phase absorption spectra we record are unambiguously associated with the chosen *m*/*z* selected ion. This situation is quite distinct from solution‐phase UV‐VIS spectroscopy, where overlapping spectra of distinctive chemical species may be present. We find that [MnL(CO)_3_(CH_3_CN)]⋅H^+^ displays strong photodepletion in the UV region above 4.4 eV (280 nm), with a sharp onset around 4.2 eV (290 nm), consistent with the known gaseous spectra of neutral and protonated Tryptophan.^48^, ^49^ (Deprotonated Tryptophan displays an onset to its first electronic excited at 315 nm.^50^)

Electronic excitations of the Mn complex within this region can therefore be linked to predominantly Tryptophan‐localized transitions associated with S_0_‐S_1_, S_0_‐S_2_ and S_0_‐S_3_.^46^ An additional, lower‐intensity region of absorption is present between 2.5–3.9 eV (490‐320 nm), peaking at ≈3 eV (415 nm). Absorption in this region is expected to correspond to metal‐to‐ligand charge‐transfer (MLCT) transitions,^19^, ^51^, ^52^ with recent TDDFT calculations indicating that ligand‐to‐metal charge‐transfer (LMCT) transitions are present.^25^

Figure 4 b displays the gaseous absorption spectrum of [MnL(CO)_3_]⋅H^+^, which again shows strong absorption in the UV region, with a sharp increase in photodepletion above 4.3 eV (290 nm). While both complexes have absorption bands which peak around 3 eV (415 nm), [MnL(CO)_3_]⋅H^+^ displays additional absorption bands compared to [MnL(CO)_3_(CH_3_CN)]⋅H^+^ which peak at 2.8 and 3.8 eV (443 and 327 nm). Figure 4 c shows the solution‐phase absorption spectrum of TryptoCORM for comparison with the gaseous spectra. (Although the protonated forms of TryptoCORM will be minor constituents in solution, pH has little effect on the UV‐VIS spectrum. Section S7 provides further information.) All three spectra clearly display similar absorptions in the UV region of the strong Tryptophan‐localized transition, with the sharp onset at 4.3 eV (290 nm) being red shifted to 4.0 eV (310 nm) in solution.^53^

### Photo‐induced loss of CO from [MnL(CO)_3_(CH_3_CN)]⋅H^+^ and [MnL(CO)_3_]⋅H^+^

To gain insight into photo‐induced loss of CO, we inspected the cationic photofragments as a function of laser excitation wavelength. [MnL(CO)_3_(CH_3_CN)]⋅H^+^ and [MnL(CO)_3_]⋅H^+^ were observed to photofragment into a number of distinctive channels, and these are discussed in more detail in the next section. Since we are primarily interested in photo‐triggered CO loss from TryptoCORM, we focus here on the major (most intense) photoinduced loss of CO channels, that is, pathways [1b] and [1c] for [MnL(CO)_3_(CH_3_CN)]⋅H^+^, and pathway [1b] for [MnL(CO)_3_]⋅H^+^. (Pathway [1b] refers to the product without the labile CH_3_CN for [MnL(CO)_3_]⋅H^+^.)

**Figure 5:**
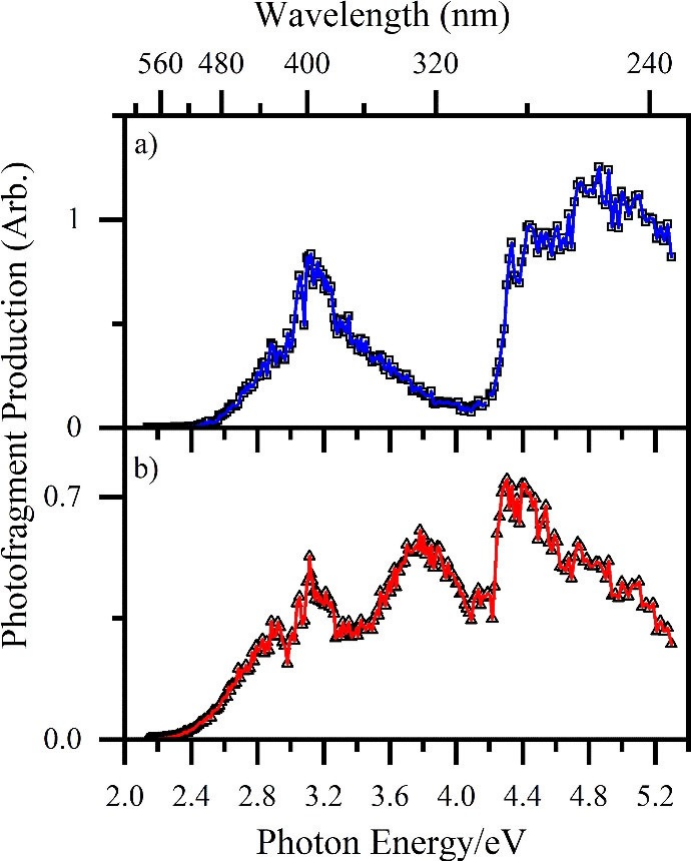
Photofragment production spectra corresponding to loss of 3CO molecules from a) [MnL(CO)_3_(CH_3_CN)]⋅H^+^ (pathways [1b] and [1c]) and b) [MnL(CO)_3_]⋅H^+^ (pathway [1b]) from 2.1–5.3 eV. See text for details.

Comparison of the Figure 5 photofragment production spectra with the gaseous absorption spectra (Figure 4) clearly shows that the propensity to eject CO closely mirrors the absorption spectra. There are several additional points of note. CO ejection from [MnL(CO)_3_]⋅H^+^ is significant over a greater wavelength range than for [MnL(CO)_3_(CH_3_CN)]⋅H^+^. While this is most evident over the region from 380–300 nm, it is also significant in the key region between 500–450 nm. Importantly, the cross section for loss of 3CO falls by around 50 % on going from 400–440 nm for [MnL(CO)_3_(CH_3_CN)]⋅H^+^ but the reduction is less for [MnL(CO)_3_]⋅H^+^ due to the increased electronic absorption in the region. The primary CO production spectra therefore clearly show that incorporation of the labile CH_3_CN ligand into TryptoCORM optimizes light‐triggered CO release further into the redder wavelength region. Taken together, the CO production spectra (Figures 5 a and 5b) also show that there is little enhancement in CO production on moving from 400 nm to bluer wavelengths such as 330 nm, and indeed total CO production dips on going through the 300 nm region.

### Photodissociation pathways of [MnL(CO)_3_(CH_3_CN)]⋅H^+^ and [MnL(CO)_3_]⋅H^+^

**Figure 6:**
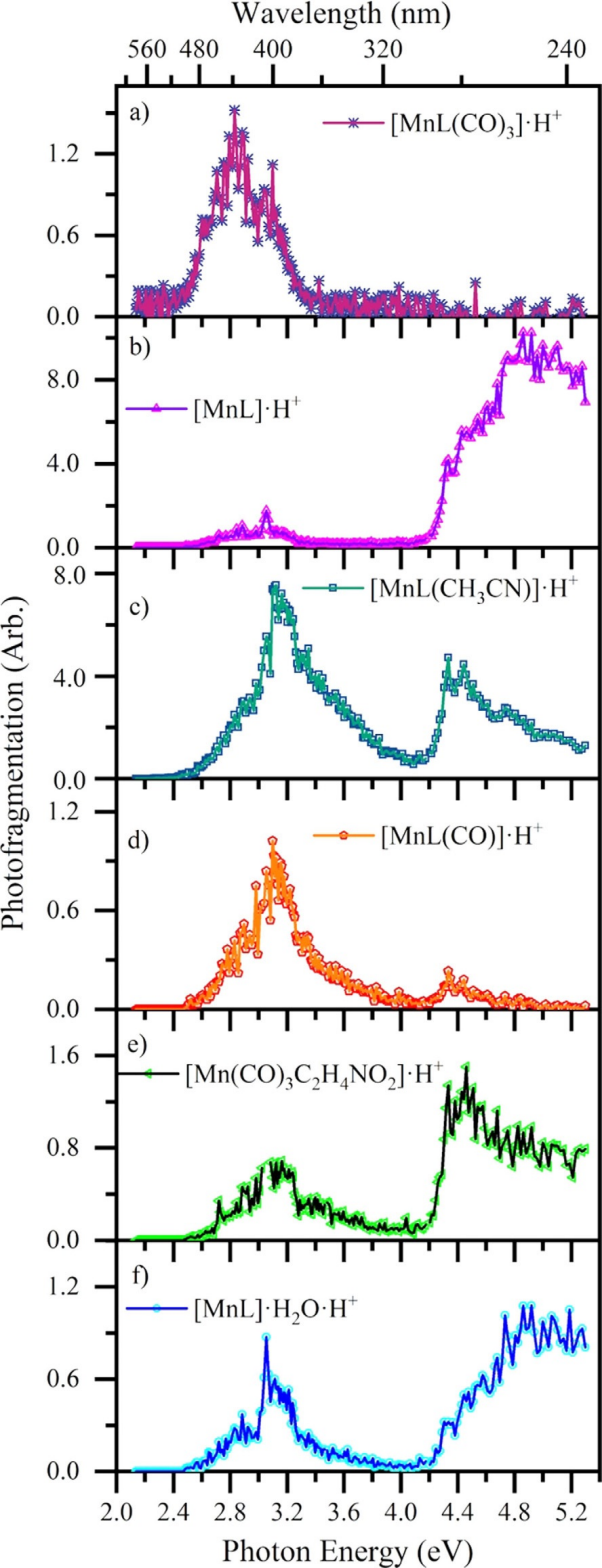
Photofragment production spectra from [MnL(CO)_3_(CH_3_CN)]⋅H^+^ across the range 2.1–5.3 eV, showing production of (a) [MnL(CO)_3_]⋅H^+^, (b) [MnL]⋅H^+^, (c) [MnL(CH_3_CN)]⋅H^+^, (d) [MnL(CO)]⋅H^+^, (e) [Mn(CO)_3_(C_2_H_4_NO_2_)]⋅H^+^, and (f) [MnL]⋅H_2_O⋅H^+^. The solid lines join the experimental data points.

Figure 6 presents the photofragment production spectra of the most intense photoions from [MnL(CO)_3_(CH_3_CN)]⋅H^+^ across 2.1–5.3 eV (234–580 nm), which correspond to the same pathways observed in HCD (Figure 3).

Photoinduced loss of the labile CH_3_CN ligand [1a], is seen only at low excitation energies between 2.4–3.2 eV (Figure 6 a), with the carbonyl loss channels dominating at higher energies.

Loss of CH_3_CN along with three COs [1c] (Figure 6 b), is one of the most intense channels, with [MnL]⋅H^+^ being produced very strongly within the high‐energy region, especially above 4.2 eV (295 nm). The second dominant photofragmentation channel [1b] (Figure 6 c) results in strong production of [MnL(CH_3_CN)]⋅H^+^ particularly over the low‐energy region. This product ion is striking as it is unanticipated on purely energetic grounds, given that HCD demonstrated that CH_3_CN is more readily lost compared to CO. As in the HCD experiment, no photofragment corresponding to ejection of a single CO is observed, and while loss of 2COs is observed (Figure 6 d) it is negligible at higher energies.

Intramolecular fragmentation of the Tryptophan moiety with accompanying CH_3_CN loss [1e] (Figure 6 e) is relatively strong over the high‐energy region (>4.2 eV) where Tryptophan‐localized transitions occur,^48^ but intriguingly, is also seen across the low‐energy region. The spectrum for [MnL]⋅H_2_O⋅H^+^ [1d] (Figure 6 f) mirrors that of the precursor [MnL]⋅H^+^ photofragment (Figure 6 b) as expected for water addition to a reactive photofragment.^54^ We note finally that the overall profile of the photodepletion spectrum can be readily recovered by summing the various photofragmentation channels. In this context, it is notable that photodepletion and photo‐fragmentation is low for [MnL(CO)_3_(CH_3_CN)]⋅H^+^ in the region between 3.6–4.2 eV (344–295 nm).

**Figure 7:**
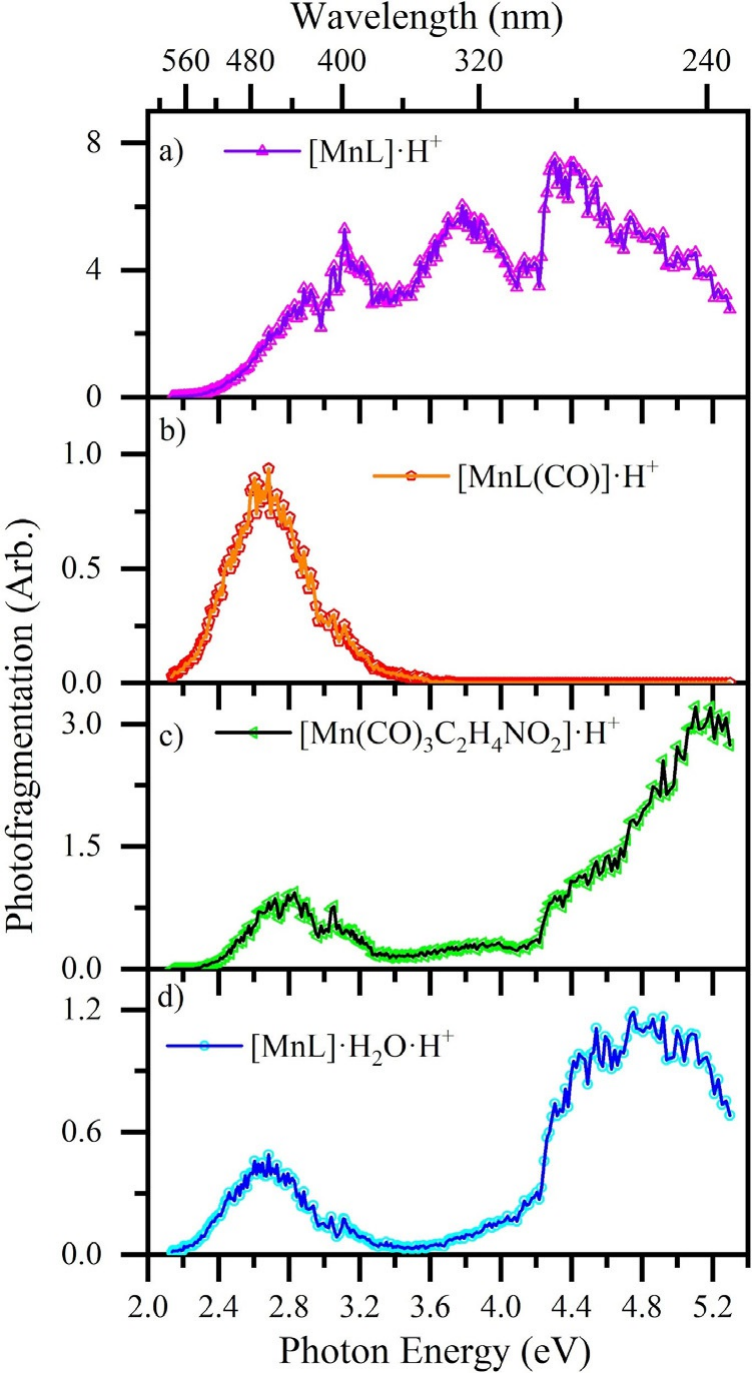
Photofragment production spectra from [MnL(CO)_3_]⋅H^+^ across the range 2.1–5.3 eV, showing production of (a) [MnL]⋅H^+^, (b) [MnL(CO)]⋅H^+^, (c) [Mn(CO)_3_C_2_H_4_NO_2_]⋅H^+^ and (d) [MnL]⋅H_2_O⋅H^+^. The solid lines join the experimental data points.

Figure 7 presents the photofragment production spectra obtained from [MnL(CO)_3_]⋅H^+^. The primary difference comparing these spectra to those of [MnL(CO)_3_(CH_3_CN)]⋅H^+^ relates to the different production profiles seen for loss of 3COs (e.g. Figure 6 b versus Figure 7 a) which were discussed in the previous section. One additional point of note is that the [MnL]⋅H_2_O⋅H^+^ production does not match that of [MnL]⋅H^+^, as it did for the same photofragments from [MnL(CO)_3_(CH_3_CN)]⋅H^+^. In particular, [MnL]⋅H_2_O⋅H^+^ is not produced to any significant extent from 3.2–4.0 eV (387–310 nm). This is an important point as it indicates that the [MnL]⋅H^+^ photofragment is not the same electronic species across the entire excitation range.

## Discussion

### Further discussion of TryptoCORM's photofragmentation profiles

**Figure 8:**
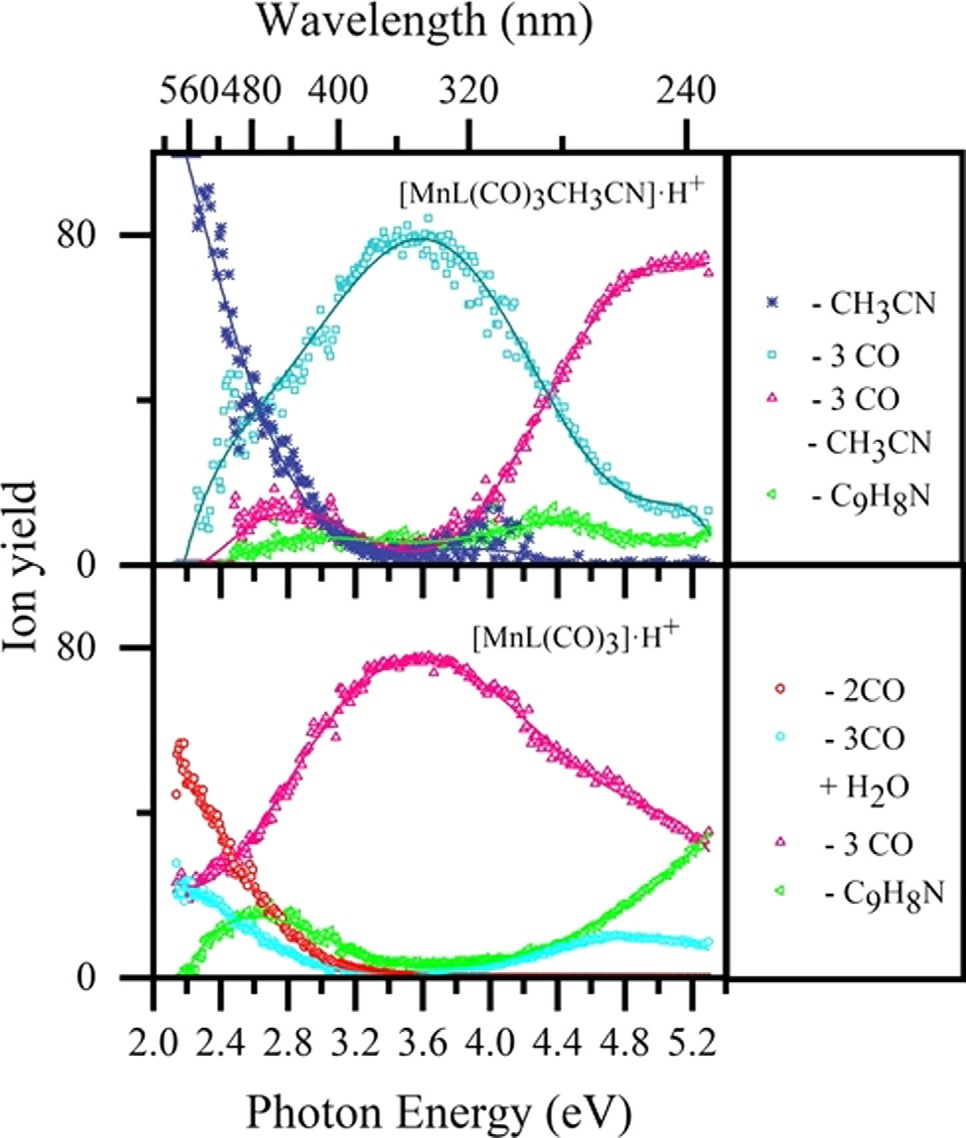
Quantum ion yield plots (in %) for photoexcitation of (a) [MnL(CO)_3_(CH_3_CN)]⋅H^+^ and (b) [MnL(CO)_3_]⋅H^+^ across the range 2.1–5.3 eV. The solid lines are five‐point adjacent averages of the data points.

An overview perspective on photofragment production is gained if we consider the quantum ion yields for the dominant photofragmentation channels of [MnL(CO)_3_(CH_3_CN)]⋅H^+^ versus [MnL(CH_3_CN)]⋅H^+^. Figure 8 displays these quantum ion yields as a function of the entire wavelength range scanned. These plots strikingly show how the photofragment production is controlled by the morphology of the excited states, and not simply by the internal energy of molecules following photoexcitation, that is, photofragment production does not simply increase as a function of photoexcitation energy. For [MnL(CO)_3_(CH_3_CN)]⋅H^+^, the dominant channel which corresponds to loss of 3COs follows a reasonably smooth bell curve shape that peaks at ≈3.6 eV (344 nm). Across the 2.9–4.8 eV (320‐258 nm) region, the 3CO loss channel for [MnL(CO)_3_]⋅H^+^ displays a very similar profile, again peaking at ≈3.6 eV (344 nm). This clearly demonstrates that both species access the same excited‐state surface across the 2.9–4.8 eV (320‐258 nm) region. At higher energies, [MnL(CO)_3_(CH_3_CN)]⋅H^+^ also increasingly loses its acetonitrile, while [MnL(CO)_3_]⋅H^+^ displays an increased propensity to lose the C_9_H_8_N unit. Both of these channels reflect the increasing internal energy of the complexes as a function of photoexcitation.

The tryptophan C_α_‐C_β_ bond rupture fragment is characteristic of the dominant photofragment that would be expected from gaseous protonated Tryptophan.^55^ While these excitations should dominate in the region above 4.3 eV (290 nm), the fact that the ‐C_9_H_8_N loss photofragment is observed in the lower‐energy bands is evidence that strong coupling occurs throughout the excitation range studied here. Indeed, this observation is in line with recent theoretical work from Fumanal et al. which has shown that the central metal atom plays a controlling role in the early time photophysics of transition metal complexes.^56^ In their studies of a manganese (I) complex, ultrafast decay of the absorbing MLCT S_2_ state was found to be mediated by vibronic coupling between the S_2_/S_1_ and the upper singlet metal‐centered and MLCT states. Crucially, in the absence of strong spin‐orbit coupling, S_2_→S_1_ internal conversion was found to be indirect and mediated by distinctive upper electronic states. This early time photophysics prepares the complex for subsequent carbonyl dissociation.

The quantum ion yield plots provide clear evidence for the presence of three distinctive excitation regions for [MnL(CO)_3_(CH_3_CN)]⋅H^+^ and [MnL(CO)_3_]⋅H^+^. The first occurs between 2.2–2.6 eV (564–413 nm), the second from 2.6–4.4 eV (413–295 nm), and the third from 4.4–5.3 eV (295–234 nm). The locations of the regions are remarkably similar for the two complexes. High‐level calculations of the dissociative excited state surfaces are now highly desirable to test how accurately the computed excited state surfaces map onto these quantum yield plots.

### Implications for PhotoCORM activity of TryptoCORM

Next, we turn to discussing our measurements of the direct photodecay pathways of isolated TryptoCORM in the context of its solution‐phase behavior. Initial myoglobin‐based spectroscopic assays of TryptoCORM indicated that the molecule did not release significant amounts of CO in the dark, but released 2 molar equivalents of CO upon irradiation at 400 nm (3.1 eV).^24^ However, subsequent work with leghemoglobin revealed that TryptoCORM did in fact release CO in the dark. This seeming contradiction was explained as resulting from differences in the binding constant of CO to the molecule it was released from versus its binding constants to myoglobin and leghemoglobin.^24^ This example highlights one of the known problems of using myoglobin assays to measure CO release.^31^ The technique demonstrated here provides a new approach to determining the wavelength dependence of CO release from a potential photoCORM in a straightforward and unambiguous manner. While there will be known differences between the gaseous and solution‐phase CO dissociation profile,^16^ our results show that for TryptoCORM, lower molar equivalents of CO are produced (>410 nm in the gas phase) in the visible region compared to the UV (<400 nm in the gas phase). This result is entirely in line with solution‐phase irradiation measurements.^24^ It is important to acknowledge that these observations do not imply that we believe that the gaseous dissociation mechanism mirrors that in solution. The mechanisms of metal carbonyl photodissociation in solution are known to differ from the gas‐phase.^57^, ^58^ For example, a metal carbonyl will typically eject a higher number of carbonyl ligands following UV/VIS excitation in the gas‐phase compared to solution, since vibrational relaxation of the excess energy to the solvent bath is not available for gaseous systems.^58^ Transient infrared spectroscopy of organometallic species in the gas phase has also provided a number of propensity rules for linking those older gas‐phase measurements with more recent solution‐phase measurements. For example, it was observed that in the gas phase, that both the coordinatively unsaturated photofragment and the ejected ligand tend to be produced with more internal energy as the energy of the photolysis photon increases, and also that the nature of the electronic state accessed can influence branching ratios for products.^58^

Ultra‐fast time‐resolved infra‐red spectroscopy has very recently been used to demonstrate that, in solution, CO‐dissociation proceeds to give ^3^[MnL(CO)_2_(CH_3_CN)] as the dominant process on irradiation at 400 nm.^25^ This occurs in under 1 ps and is followed by a change in spin and coordination of the solvent, S, to give ^1^[MnL(CO)_2_(CH_3_CN)(S)] (*τ*≈20 ps). Compared to the solution‐phase photolysis measurements which were conducted with two fixed wavelength diode LEDs, however, our photolysis experiment incorporates a broad scanning laser source. We therefore obtain a full picture of the spectral regions where CO loss is maximized via loss of three CO units from the TryptoCORM, as shown in exquisite detail on the quantum ion‐yield plots of Figure 8. Perhaps just as importantly, these plots allow us to trace the extent to which maximum CO loss is compromised at the redder wavelengths, and hence make an informed decision as to the optimum wavelength for clinical application.

## Conclusions

In conclusion, we have measured the intrinsic absorption spectrum and photofragmentation pathways for TryptoCORM as an isolated molecular complex in the gas‐phase. The photofragment production spectra reveal the optimum excitation wavelengths for maximizing CO ejection from a photoCORM for the first time, and therefore demonstrate a straightforward and widely applicable new methodology for assessing photoinduced CO release. Importantly, our gas‐phase results can be directly linked to the solution‐phase properties of the system by comparing the band positions of the gaseous and solution‐phase absorption spectra. The experiments reported here result from applying novel laser‐interfaced mass spectrometry within an adapted commercial mass spectrometer, a technique that has considerable broader potential for applied photochemical and photophysical studies.^27^, ^59^ In particular, the experiments performed here are not limited to photoCORMs but could be readily applied to other photo‐activated prodrugs including platinum anticancer complexes and H_2_S or CS_2_ releasing therapeutical agents.^60^, ^61^, ^62^ Finally, we note that our results also represent the first direct gas‐phase measurement of the wavelength‐dependent photochemical branching ratios (quantum ion yields) for a transition metal carbonyl complex. The spectra presented here therefore provide a new benchmark against which high‐level excited state calculations of this challenging group of molecular systems can be compared.

## Experimental Section

**Cryogenic ion vibrational spectroscopy**: The gas‐phase infrared photodissociation spectra presented here were obtained in a custom‐built spectrometer described in detail elsewhere.^38^ Briefly, solutions of TryptoCORM in CH_3_CN (≈10^−3^ m) with trace amounts of formic acid were electrosprayed to generate [MnL(CO)_3_]⋅H^+^ ions, where the L ligand is deprotonated Tryptophan. Ions were directed to a 3D quadrupole ion trap held at 10 K by a closed‐cycle helium cryocooler, and D_2_‐tagged adducts, that is, [MnL(CO)_3_]⋅H^+^⋅D_2_, were then produced by introducing a ≈1 ms burst of He, seeded with 10 % D_2_. The [MnL(CO)_3_]⋅H^+^⋅D_2_ aggregates were then ejected into the time‐of‐flight mass spectrometer, mass selected via a gated deflector, and intersected with the output of a Nd:YAG pumped tunable OPO/OPA laser between the ranges 1400–2300 and 2400–3800 cm^−1^. Resonant absorption of one IR photon leads to loss of the D_2_ tag, that is, [Eq. (1)]

(1) $$\left[\mathrm{MnL}\left(\mathrm{CO}\right){}_{3}\right]\cdot H{}^{+}\cdot D{}_{2}+h\nu {}_{\mathrm{IR}}\to \left[\mathrm{MnL}\left(\mathrm{CO}\right){}_{3}\right]\cdot H{}^{+}+D{}_{2}$$

with absorption being monitored by measuring the intensity of the resulting photofragment, [MnL(CO)_3_]⋅H^+^, as a function of photon wavelength. This represents the gas‐phase IR spectrum.

**Higher‐energy collisional dissociation**: Higher‐energy collisional dissociation (HCD) was performed to investigate the ground‐state thermal fragmentation characteristics of [MnL(CO)_3_(CH_3_CN)]⋅H^+^ and [MnL(CO)_3_]⋅H^+^, using an Orbitrap™ Fusion Tribrid mass spectrometer (Thermo Fisher Scientific, Waltham, MA, U.S.A.) as described previously.^44^ HCD energies from 0–30 % HCD were employed. Solutions of TryptoCORM (1×10^−5^ mol dm^−3^) in H_2_O:CH_3_CN 1:1 were introduced to the mass spectrometer (for HCD and LIMS) through electrospray ionization using a capillary temperature of 140 °C.

**Laser‐interfaced mass spectrometry**: Gas‐phase UV photodissociation experiments were conducted in a laser‐interfaced amaZon ion‐trap mass spectrometer (LIMS), which was modified as described in detail elsewhere.^26^, ^63^ The LIMS instrument has all the advantages of a commercial mass spectrometer (e.g. flexible ion sources, mass selection and isolation of primary and secondary ions and fragments via MS^n^ schemes), coupled with the ability to record UV absorption and photofragmentation spectra in a routine manner. The UV photons in these experiments were produced by an Nd:YAG (10 Hz, Surelite) pumped OPO (Horizon) laser, giving ≈1 mJ across the range 2.1–5.3 eV (234–580 nm). A laser step size of 1 nm was employed for all scans, with photofragmentation experiments being run with an ion accumulation time of 100 ms. A fragmentation time of 100 ms was employed, so that each mass‐selected ion packet interacted with one laser pulse to minimize multiphoton excitation. Laser power studies were conducted at 400 and 280 nm to verify that only a single photon is required to induce photofragmentation.

Photodepletion intensity (PD) and photofragment production (PF) were calculated using Equations (2), (3):

(2) $$\mathrm{Photodepletion}\;\mathrm{Intensity}\;=\;\frac{\ln(\frac{{\mathrm{Int}}_{\mathrm{OFF}}}{{\mathrm{Int}}_{\mathrm{ON}}})}{\lambda \;\times \;P}$$

(3) $$\mathrm{Photofragmentation}\;\mathrm{Intensity}\;=\;\frac{\left(\frac{{\mathrm{Int}}_{\mathrm{FRAG}}}{{\mathrm{Int}}_{\mathrm{OFF}}}\right)}{\lambda \;\times \;P}$$

where Int_ON_ and Int_OFF_ are the peak intensities with laser on and off, Int_Frag_ the fragment intensity with laser on, λ the excitation wavelength (nm) and P the laser pulse energy (mJ). The photodepletion spectrum is considered to be equivalent to the gaseous absorption spectrum in the limit where fluorescence is negligible. Quantum ion yields are calculated according to Equation (4):

(4) $$\mathrm{Ion}\;\mathrm{Yield}=\mathrm{Int}{}_{\mathrm{FRAG}}/\Sigma \;\mathrm{Int}{}_{\mathrm{PFT}}$$

where Int_PFT_ is the sum of the photofragment ion intensities obtained with the laser on.

**TryptoCORM synthesis**: d‐TryptoCORM was synthesized following a previously published protocol.^24^

**Quantum chemistry calculations**: Density Functional Theory (DFT) calculations were performed in Gaussian 09,^64^ using standard methods. Full details are given in Sections S2 and S3, with optimized geometries in Section S4.

## Conflict of interest

The authors declare no conflict of interest.

## Supporting information

As a service to our authors and readers, this journal provides supporting information supplied by the authors. Such materials are peer reviewed and may be re‐organized for online delivery, but are not copy‐edited or typeset. Technical support issues arising from supporting information (other than missing files) should be addressed to the authors.

Supplementary
