# Supplementary material for: Direct Measurement of the Visible to UV Photodissociation Processes for the PhotoCORM TryptoCORM
Source: Chemistry. 2020 Jul 24;26(45):10297–306. doi: 10.1002/chem.202001077 (PMC7496620; doi:10.1002/chem.202001077)
Supplement: Supplementary file 1 — Supplementary [file CHEM-26-10297-s001.pdf]

Chemistry–A European Journal

Supporting Information

Direct Measurement of the Visible to UV Photodissociation Processes for the PhotoCORM TryptoCORM**

Rosaria Cercola,[a] Kaitlyn C. Fischer,[b] Summer L. Sherman,[b] Etienne Garand,[b] Natalie G. K. Wong,[a] L. Anders Hammerback,[a] Jason M. Lynam,[a] Ian J. S. Fairlamb,[a] and Caroline E. H. Dessent*[a]

Author Contributions

C.D. Conceptualization: Lead; Formal analysis: Equal; Funding acquisition: Lead; Methodology: Lead; Project administration: Lead; Resources: Lead; Supervision: Lead; Writing - Original Draft: Lead
R.C. Data curation: Lead; Formal analysis: Lead; Investigation: Lead; Writing - Original Draft: Supporting; Writing - Review & Editing: Supporting
K.F. Data curation: Supporting; Formal analysis: Supporting; Investigation: Supporting; Writing - Review & Editing: Supporting
S.S. Data curation: Supporting; Formal analysis: Supporting; Investigation: Supporting; Writing - Review & Editing: Supporting
E.G. Formal analysis: Supporting; Funding acquisition: Equal; Methodology: Supporting; Resources: Equal; Writing - Review & Editing: Supporting
N.W. Data curation: Supporting; Investigation: Supporting; Writing - Review & Editing: Supporting
L.H. Investigation: Supporting
J.L. Formal analysis: Supporting; Funding acquisition: Supporting; Resources: Supporting; Supervision: Supporting; Writing - Review & Editing: Supporting
I.F. Conceptualization: Supporting; Funding acquisition: Supporting; Resources: Supporting; Supervision: Supporting; Writing - Review & Editing: Supporting.

Table of Contents

S1. Electrospray ionization mass spectrum of TryptoCORM
S2. Computational study of the geometric structures of $MnL(CO)_3(CH_3CN)$ and its protomer $[MnL(CO)_3(CH_3CN)]\cdot H^+$
S3. Computational study of the geometric structures of $[MnL(CO)_3]\cdot H^+$
S4. IR-IR conformer-specific spectroscopy of $[MnL(CO)_3]\cdot H^+$
S5. Thermal fragmentation pathways of $[MnL(CO)_3(CH_3CN)]\cdot H^+$ and $[MnL(CO)_3]\cdot H^+$
S6. Comparison of thermal and photofragmentation pathways
S7. Solution-phase UV-VIS spectra of TryptoCORM as a function of pH

S1. Electrospray Ionization mass spectrum of TryptoCORM

Figure S1 displays the positive ion mode electrospray ionization mass spectrum of TryptoCORM. The protonated pseudo molecular ion, $[MnL(CO)_3(CH_3CN)]\cdot H^+$, where L denotes the deprotonated tryptophan ligand, appears strongly at *m/z* 384. The most intense ion has *m/z* 343 and corresponds to the protonated molecular species without the CH_3CN ligand, *i.e.* $[MnL(CO)_3]\cdot H^+$.

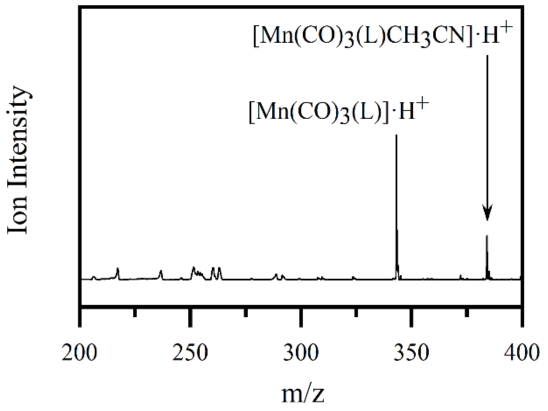

Figure S1. Positive ion mode electrospray ionization mass spectrum of TryptoCORM in CH_3CN (10^{-5} M with 0.1% TFA).

S2. Computational study of $MnL(CO)_3(CH_3CN)$ and $[MnL(CO)_3(CH_3CN)]\cdot H^+$ geometry

To explore the possible geometric structures adopted by $[MnL(CO)_3(CH_3CN)]\cdot H^+$, we first optimized a range of geometric structures of $MnL(CO)_3(CH_3CN)$. Geometric optimizations were performed in Gaussian 09 at the PBE0/Def2SV level,[1] with frequency calculations being conducted to test that the structures obtained corresponded to local minima. Geometric isomers of TryptoCORM with a L-Tryptophan identified previously by Ward et al. were used as starting points for these calculations.[2] (The calculations displayed below have been performed on TryptoCORM with a D-Tryptophan, as used in the current experimental work). Table S1 displays the structures obtained along with their relative energies (non-zero point energy corrected).

Protons were added to the most favourable protonation sites of $MnL(CO)_3(CH_3CN)$ (labelled 1-3 in Scheme 1 of the main text) to obtain starting structures for $[MnL(CO)_3(CH_3CN)]\cdot H^+$. The lowest-energy optimized resulting structures are displayed in Table S2, along with their relative energies (non-zero point energy corrected).

Table S1. Optimized structures (PBE0/Def2SV) of $MnL(CO)_3(CH_3CN)$. Relative energies (kJ mol^{-1}) in the gas-phase and in CH_3CN solvent (in parentheses) are shown.[a]

[A2]	[B1]	[C2]
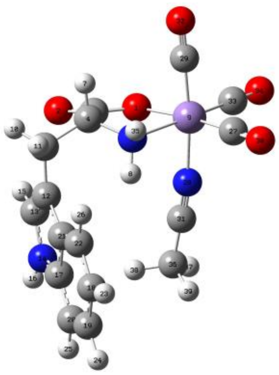	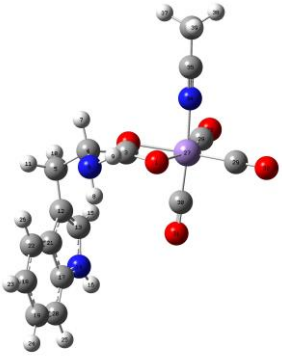	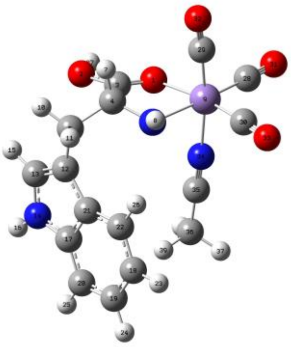
0	33.8	137
(0)	(54.7)	(173)
[A1]	[B2]	[C1]
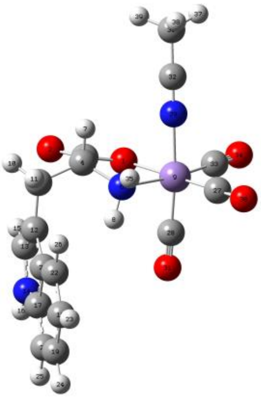	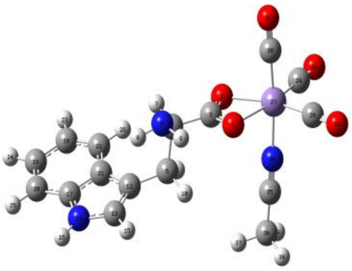	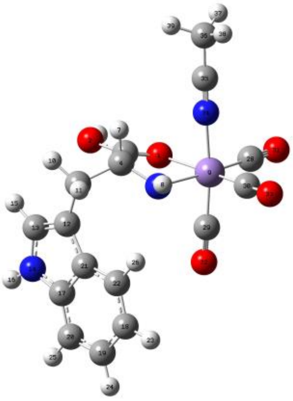
8.36	46.6	160
(4.87)	(60.3)	(165)

[a] Labels in square brackets follow assigned isomer names from Ref. [2].

Table S2. Optimized structures (PBE0/Def2SV) of $[MnL(CO)_3(CH_3CN)]^{\cdot}H^+$. Relative energies (kJ mol^{-1}) in the gas-phase and in CH_3CN solvent (in brackets) are shown.

[A2_O1]	[A2_O2]	[A1_O1]
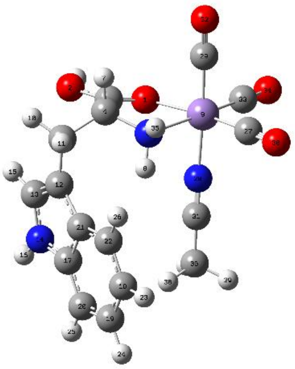	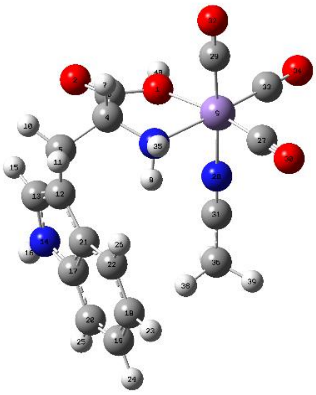	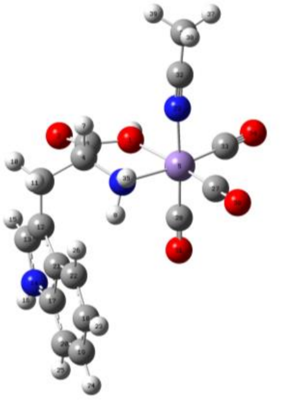
0	34.0	42.0
(0)	(22.6)	(31.7)

[A1_O1]	[B1_N3]	[B2_N3]
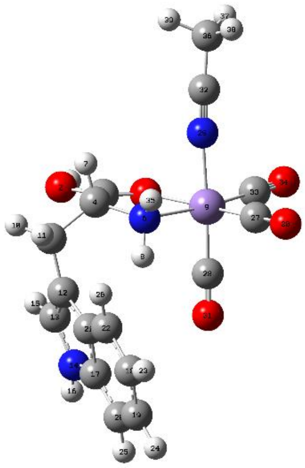	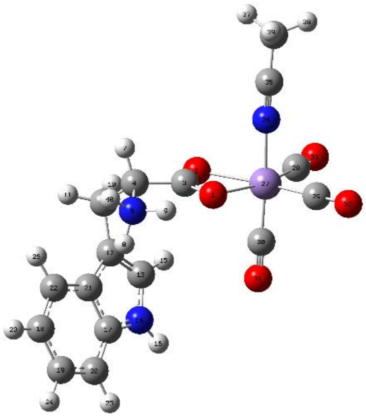	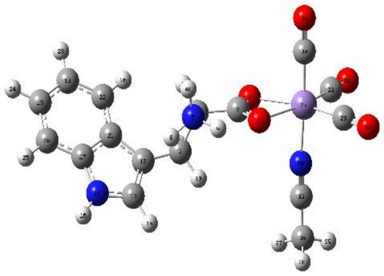
6.92	35.7	47.2
(4.51)	(27.6)	(23.8)

[a] The first part of the labels in square brackets follow assigned isomer names from Ref. [2], with the second part referring to the protonation site (Scheme 1) of the main text.

S3. Computational study of [MnL(CO)$_3$]·H$^+$ geometry

To explore the possible geometric structures adopted by [MnL(CO)$_3$]·H$^+$, we removed the CH_3CN group from the MnL(CO)$_3$(CH_3CN) structures displayed in Table S1, then introduced protons at the most favourable protonation sites (labelled 1-3 in Scheme 1 of the main text) as well as directly on the Mn centre. Geometric optimizations were performed in Gaussian 09 at the PBE0/Def2SV level,[1] with frequency calculations being conducted to test that the structures obtained corresponded to local minima. Table S3 displays the resulting lowest-energy structures, along with their relative energies (non-zero point energy corrected). These structures include several where the indole ring coordinates directly to the vacant metal site (*i.e.* “folded” structures). Table S4 shows key distances between the Mn and the indole ring in the folded structures. Finally, Table S5 presents the gas-phase and solution-phase Boltzmann populations for the structures presented in Table S3.

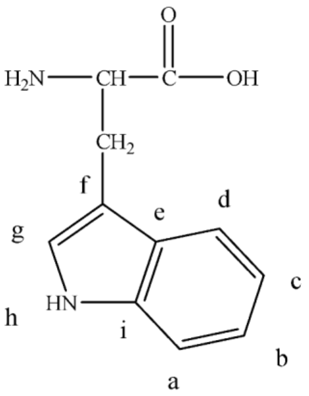

Scheme S1. Schematic diagram of tryptophan, with atom labels on the indole ring.

Table S3. Optimized structures (PBE0/Def2SV) of $[MnL(CO)_3]^{\cdot}H^+$.[a] Relative energies (kJ mol^{-1}) in the gas-phase and in CH_3CN solvent (in brackets) are shown.

[A2_O1_Folded]	[B1_N3_Open]	[A2_O2_Open]
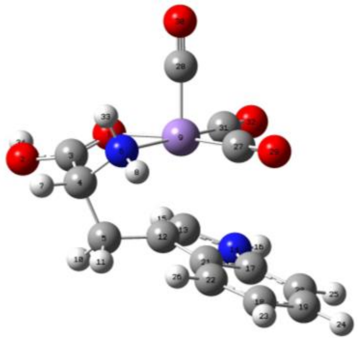	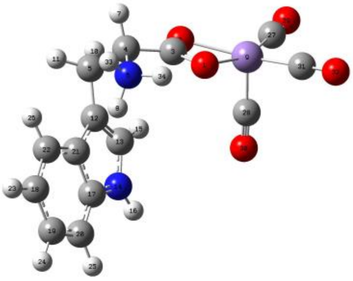	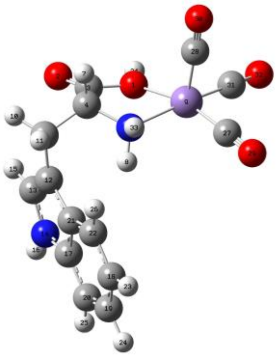
0 (0)	69 (48.2)	85 (60.9)
[A2_O2_Folded]	[B2_N3_Folded]	[C2_O2_Folded]
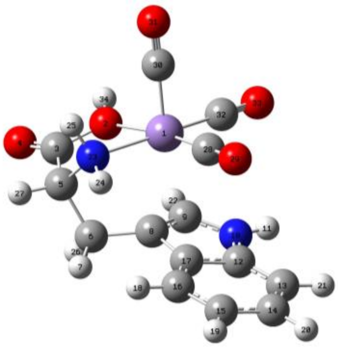	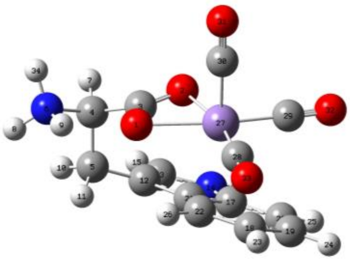	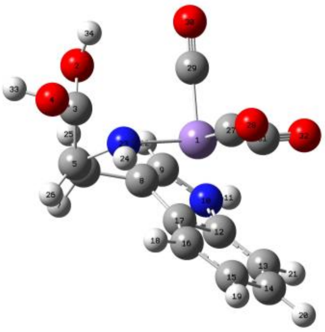
40.5 (29.5)	71.2 (44.)	184 (200)
[A2_O1_Open]	[B2_N3_Open]	[C1_MnH_Open]
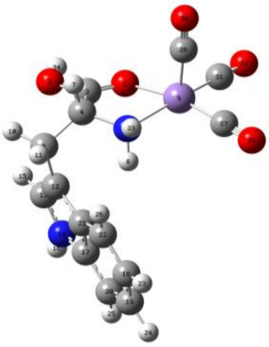	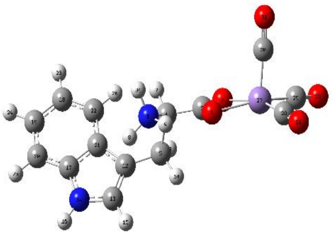	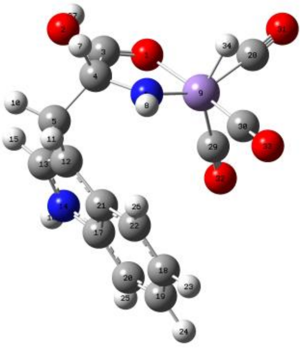
44.6 (30.3)	75.7 (48.9)	221 (238)

[a] The first part of the labels in square brackets follow assigned isomer names from Ref. [2], with the second part referring to the protonation site (Scheme 1 of the main text). Folded refers to a structure where the metal centre closely coordinates with tryptophan, while open refers to a structure where the metal centre has a vacant coordination site.

Table S4. Distances between Mn and C and H atoms on the indole ring in the folded structures of $[MnL(CO)_3]\cdot H^+$, with atom labels following Schemes 1 and S1. Only distances less than 3 Å are shown.

Structure	Bond	Bond Length (Å)
A2_O1_Folded	Mn-C_f	2.5
	Mn-C_g	2.6
	Mn-H_g	3.0

A2_O2_Folded	Mn-C_f	2.5
	Mn-C_g	2.5
	Mn-H_g	3.0
B2_N3_Folded	Mn-C_d	2.6
	Mn-H_d	2.7
	Mn-C_e	2.9
C2_O2_Folded	Mn-C_e	2.3
	Mn-C_f	2.4
	Mn-C_d	2.6
	Mn-H_d	2.9

Table S5. Boltzmann populations of the isomers of $[MnL(CO)_3]\cdot H^+$ at 373 K.

Structure	Gas-phase Boltzmann Population	Solution-phase Boltzmann Population
A2_O1_Folded	99.99	99.99
A2_O2_Folded	$2.28*10^{-4}$	$7.77*10^{-3}$
A2_O1_Open	$6.13*10^{-5}$	$6.02*10^{-3}$
B1_N3_Open	$2.45*10^{-8}$	$1.93*10^{-5}$
B2_N3_Folded	$1.21*10^{-8}$	$7.43*10^{-5}$
B2_N3_Open	$2.85*10^{-9}$	$1.54*10^{-5}$
A2_O2_Open	$1.44*10^{-10}$	$3.29*10^{-7}$
C2_O2_Folded	$2.34*10^{-24}$	$1.38*10^{-26}$
C1_MnH_Open	$1.64*10^{-29}$	$7.03*10^{-32}$

S4. IR-IR conformer-specific spectroscopy of $[MnL(CO)_3]\cdot H^+$

The number of isomeric species contributing to the IR spectrum of $[MnL(CO)_3]\cdot H^+$ was determined by performing IR-IR conformer-specific spectroscopy. This involves the use of a second IR laser (pump) which is introduced at the same frequency of one of the observed spectral lines for a given species, thereby reducing the ground state population associated with that species. If the spectrum is then reacquired with the IR probe laser, all spectral lines associated with the species giving rise to the spectral line that is hole-burnt will be reduced in intensity. In the current measurements, the pump laser was a second Nd:YAG pumped OPO/OPA laser, which was focused to the centre of the 3D ion trap ~ 90 ms after the introduction of the buffer gas.[3]

IR-IR conformer-specific spectra were obtained at 3347, 3494 and 3547 cm^{-1},[4] and are displayed in Figure S2. Hole-burning at these frequencies leads to an overall decrease in the general intensity of the entire spectrum, in line with the presence of just a single structural isomer. Although multiple rotamers may be present, with very similar IR absorption profiles, these are unlikely to display differing photochemical properties.

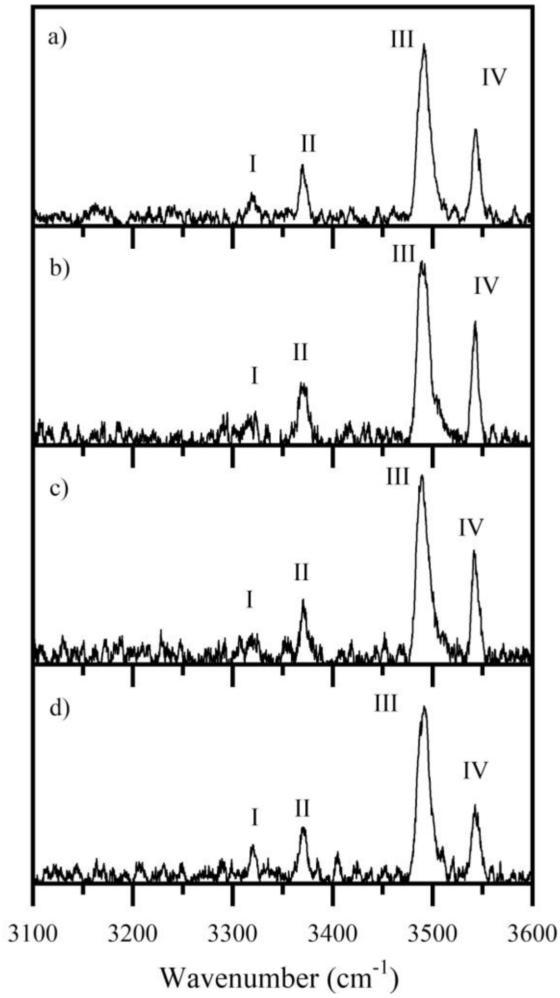

Figure S2. (a) The gas-phase IR spectrum of $[Mn(CO)_3(L)]\cdot H^+$, with IR-IR conformer specific spectra obtained with the hole-burning laser set to (b) 3347, (c) 3494, and (d) 3547 cm^{-1}.

S5. Thermal fragmentation pathways of $[MnL(CO)_3(CH_3CN)]{\cdot}H^+$ and $[MnL(CO)_3]{\cdot}H^+$

Prior to performing laser photodissociation, we investigated the thermal fragmentation pathways of $[MnL(CO)_3(CH_3CN)]{\cdot}H^+$ and $[MnL(CO)_3]{\cdot}H^+$ by performing higher-energy collisional dissociation (HCD). This experiment maps out the fragmentation pathways as a function of internal energy,[5] and is therefore also of interest in the context of the known propensity of TryptoCORM to release CO upon thermal exposure, *i.e.* to behave as a thermal-CORM.[6,7]

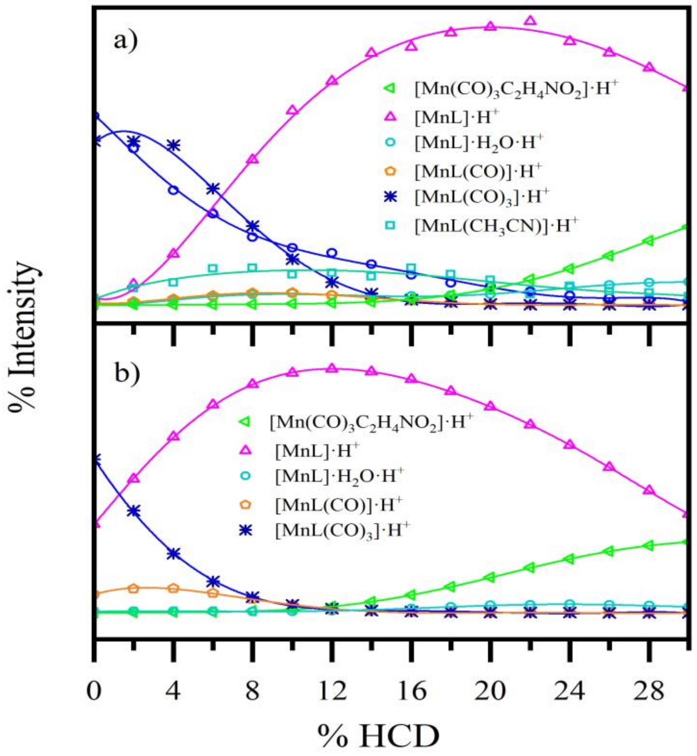

Figure S3. Precursor ion decay curves and fragment ion production curves for (a) $[MnL(CO)_3(CH_3CN)]{\cdot}H^+$ and (b) $[MnL(CO)_3]{\cdot}H^+$ upon HCD between 0-30%.

Figure S3a displays the HCD fragmentation curves for $[MnL(CO)_3(CH_3CN)]{\cdot}H^+$ which correspond to the following processes:

$[MnL(CO)_3(CH_3CN)]{\cdot}H^+ \rightarrow [MnL(CO)_3]{\cdot}H^+ + CH_3CN$ [1a]

$[MnL(CO)_3(CH_3CN)]{\cdot}H^+ \rightarrow [MnL(CO)_3]{\cdot}H^+ + CH_3CN \rightarrow [MnL]{\cdot}H^+ + 3CO + CH_3CN$ [1b]

$[MnL(CO)_3(CH_3CN)]{\cdot}H^+ \rightarrow [MnL(CO)(CH_3CN)]{\cdot}H^+ + 2CO$ [1c]

$[MnL(CO)_3(CH_3CN)]{\cdot}H^+ \rightarrow [Mn(CO)_3(C_2H_4NO_2)]{\cdot}H^+ + CH_3CN + C_9H_8N$ [1d]

$[MnL(CO)_3(CH_3CN)]{\cdot}H^+ \ (+ H_2O)_{trap} \rightarrow [MnL]{\cdot}H_2O{\cdot}H^+ + CH_3CN + 3CO$ [1e]

Pathway [1a] is the major, low-energy fragmentation pathway and corresponds to loss of CH_3CN. Indeed, the complex dissociates into this channel even at 0% HCD, revealing that the ion is metastable with respect to CH_3CN loss in the gas phase. The resulting $[MnL(CO)_3]{\cdot}H^+$ ion subsequently decreases in intensity across the relatively low-energy region from 0-12%, with a concomitant rise in intensity of $[MnL]{\cdot}H^+$ via pathway [1b]. Fragmentation with loss of just two CO units (pathway [1c]) is a much less intense channel, that occurs only in the lower-energy region <10%. Notably, there is no channel associated with loss of a single CO, revealing that if one CO is lost, the energetic barrier to loss of a second CO is very low. The dominance of the loss of 3COs leads us to conclude that there is also only a small barrier towards loss of 3CO units once the barrier to loss of 2CO units is reached. Similar CO loss patterns have been observed in previous CID experiments of metal carbonyl complexes, especially where structural rearrangement of the core or ligands can occur.[8,9]

The $[Mn(CO)_3(C_2H_4NO_2)]{\cdot}H^+$ fragment (pathway [1d]) increases in intensity through the high-energy collision range from 14-30% HCD. It arises from C_α-C_β homolytic dissociation bond rupture of tryptophan, as illustrated in Scheme S2. This pathway corresponds to a high-energy thermal fragmentation surface where the 3CO ligands remain bound to the Mn metal centre. Notably, we do not observe collisional-activated loss of intact tryptophan.

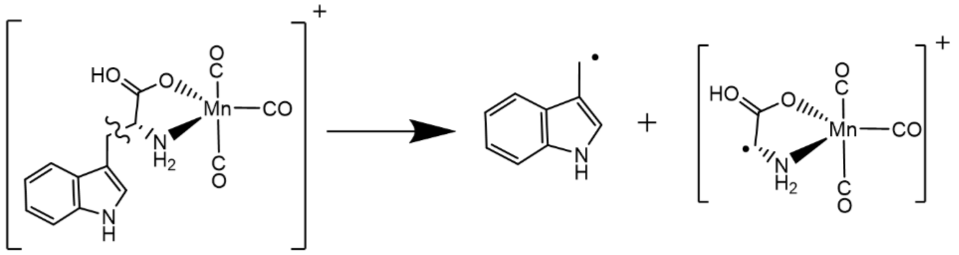

Scheme S2. Illustration of the C_{α}-C_{β} tryptophan bond rupture in $[MnL(CO)_3]\cdot H^+$.

$[MnL]\cdot H_2O\cdot H^+$ appears as a relatively low intensity fragmentation product at higher collision energies (pathway [1e]). Addition of residual water vapour to trapped ions is a known process.[10] However, the $[MnL]\cdot H_2O\cdot H^+$ fragment production profile is surprising as it does not match that of $[MnL]\cdot H^+$. This indicates that the $[MnL]\cdot H^+$ fragment does not have the same geometric and/or electronic structure when produced at different HCD energies, with only the $[MnL]\cdot H^+$ produced above 16%HCD being in a form that is readily able to bind water. It is interesting to compare these results to the ones observed when $[MnL]\cdot H^+$ is produced via photodissociation (main text, Figures 6 and 7). For the photoinduced product from $[MnL(CO)_3(CH_3CN)]\cdot H^+$ (Figure 6), the $[MnL]\cdot H_2O\cdot H^+$ production profile closely matches that of $[MnL]\cdot H^+$, suggesting that the $[MnL]\cdot H^+$ ions produced at all photon energies are able to react with water vapour. However, for $[MnL(CO)_3]\cdot H^+$, the $[MnL]\cdot H^+$ fragment production profile does not mirror those of $[MnL]\cdot H_2O\cdot H^+$ (Figure 7). These results likely reflect the electronic and geometric flexibility that is available to the fragmentation products of TryptoCORM.

Figure S3b presents the corresponding HCD data for $[MnL(CO)_3]\cdot H^+$, illustrating that the fragmentation pathways largely mirror those of $[MnL(CO)_3(CH_3CN)]\cdot H^+$, both in terms of the pathways observed and their energy dependence. (The exception here is the relative profiles of the $[MnL]\cdot H^+$ and $[MnL]\cdot H_2O\cdot H^+$ photofragments as discussed in the previous paragraph.)

S6. Comparison of thermal and photofragmentation pathways

Across the visible photoexcitation range (2.1-3.3 eV, 580-380 nm), loss of 3CO ligands from $[MnL(CO)_3(CH_3CN)]\cdot H^+$ is the major fragmentation pathway, and in striking contrast to the thermal fragmentation situation, the CH_3CN ligand largely remains attached. Loss of this CH_3CN alongside the 3COs is observed photochemically, but only as a much more minor photodecay channel. In the higher-energy UV region of the spectrum, simultaneous loss of CH_3CN + 3CO is the major photofragmentation channel, mirroring the thermal fragmentation process. However, loss of the 3CO molecules with the CH_3CN still bound to the metal centre remains a prominent photofragmentation channel across this region.

In solution, when a photoexcited species is able to relax rapidly back to the electronic ground state, the excess energy gained can be dissipated by heat loss to the bulk solvent. However, in the gas-phase, this energy is conserved within the molecular system, so that an ensuing hot ground state accessed following ultrafast decay will dissociate across the lowest-energy fragmentation barriers. These are the same fragmentation barriers that are followed when the isolated molecule is heated (as in HCD), leading to a situation termed “statistical fragmentation”. In contrast, “non-statistical” (or non-ergodic) processes occur when dissociation proceeds directly from the excited state without significant adiabatic conical-intersection involvement to return the system back to the ground state in the vicinity of the initial geometry.[5,11,12]

Earlier work from our group indicates that visible photofragmentation excitation energies (2.2-3.4 eV, 560-360 nm) will correspond to HCD energies of around 10%.[5] For $[MnL(CO)_3(CH_3CN)]\cdot H^+$, statistical decay would lead predominantly to loss of CH_3CN+3CO, while $[MnL(CO)_3]\cdot H^+$ would lose 3CO. $[MnL(CO)_3(CH_3CN)]\cdot H^+$ therefore very clearly exhibits predominantly non-statistical photofragmentation, whereas $[MnL(CO)_3]\cdot H^+$ appears to undergo statistical dissociation across the spectral region studied here.

Is it reasonable that $[MnL(CO)_3(CH_3CN)]\cdot H^+$ and $[MnL(CO)_3]\cdot H^+$ should be displaying such distinctive photophysical behaviour? In fact, the photodissociation quantum yield plots presented in Figure 8 of the main text provide evidence that the two systems exhibit remarkably similar photochemistry, given how similar the photodissociation profiles are. The probable explanation for these seeming contradictory observations is likely to lie in conformational flexibility that is available to the $[MnL(CO)_3]\cdot H^+$ moiety. While loss of 3COs via either thermal (HCD) or photo-excitation results in a $[MnL]\cdot H^+$ unit, it is not necessarily the case that this ion is the same geometric structure in the two cases. One possibility is that HCD leads to the tryptophan becoming uncoordinated from the Mn centre, whereas in the photochemically produced $[MnL]\cdot H^+$, the indole double bond of the tryptophan ligand remains attached, *i.e.* a pair of “open” and “folded” geometric structures as in Figure 1 of the main text. Such a situation would then correspond to photophysics that would be categorized as non-statistical for $[MnL(CO)_3]\cdot H^+$, fully in line with the behaviour we observe for $[MnL(CO)_3(CH_3CN)]\cdot H^+$.

Time-resolved measurements are of course necessary to characterise the photodissociation dynamics.[12] The non-statistical fragmentation pathways identified here suggest that these dynamics will be complex, as might be expected for these conformational flexible ions.

S7. Solution-phase UV-VIS spectra of TryptoCORM as a function of pH

Solution-phase UV-VIS absorption spectra of TryptoCORM (1×10^{-5} mol dm^{-3}) in MeCN/H_2O (50:50) mixtures were recorded using a UV-1800 UV-Visible spectrophotometer (Shimadzu, Kyoto, Japan) with a 10 mm UV quartz cuvette. Here, HCl (3.0 M) or (0.1 M), and formic acid (1%) were added to pH = 7.0 solutions to achieve a variety of resulting pH solutions. Formic acid was used in the first set of measurements (Figure S4a) since this acid was used to enhance production of the protonated molecular ion in electrospray (Section S1). HCl was used to record a second set of measurements for comparison. (Figure S4b). With formic acid, it was not possible to raise the solution pH above 3.75, presumably due to buffering by the formate anion. (The pK_a of tryptoCORM is thought to be around 4.5.[7])

The spectra show that the UV-VIS spectrum of tryptoCORM varies only modestly as a function of pH. Considering the spectra displayed in Figure S4b, the intense onset in absorption around 310 nm shifts slightly to the blue as pH increases from 2.5-0.5, and there is a corresponding reduction in intensity of the band in centred at 380 nm. However, these changes are small (particularly going from neutral pH to 4.5) and indicate that the key electronic transitions of tryptoCORM and its protonated analogue are similar.

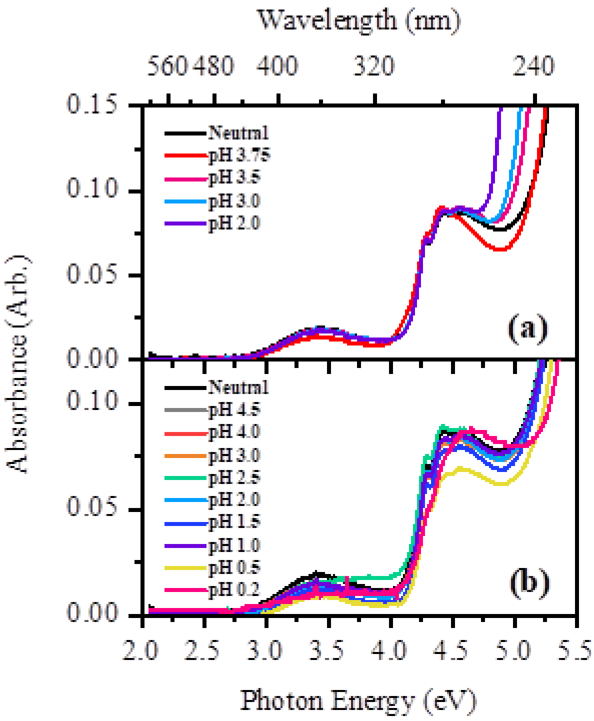

Figure S4. (a) Solution-phase absorption spectra of TryptoCORM in MeCN/H_2O (50:50) at pH 3.75-2.0, protonated with formic acid. **(b)** Solution-phase absorption spectra of TryptoCORM in MeCN/H_2O (50:50) at pH 4.5-0.2, protonated with HCl (3.0 M or 0.1 M). The absorption spectrum of TryptoCORM in its neutral form (black line) is displayed for ease of comparison.

References

[1] M. J. Frisch, G. W. Trucks, H. B. Schlegel, G. E. Scuseria, M. A. Robb, J. R. Cheeseman, G. Scalmani, V. Barone, B. Mennucci, G. A. Petersson, et al., *Gaussian 09, Rev. D.01, Gaussian Inc.* **2009**, Wallingford, CT.

[2] J. S. Ward, J. M. Lynam, J. Moir, I. J. S. Fairlamb, *Chem. - A Eur. J.* **2014**, *20*, 15061–15068.

[3] B. M. Marsh, J. M. Voss, E. Garand, *J. Chem. Phys.* **2015**, *143*, 204201.

[4] C. M. Leavitt, A. B. Wolk, J. A. Fournier, M. Z. Kamrath, E. Garand, M. J. Van Stipdonk, M. A. Johnson, *J. Phys. Chem. Lett.* **2012**, *3*, 1099–1105.

[5] R. Cercola, E. Matthews, C. E. H. Dessent, *J. Phys. Chem. B* **2017**, *121*, 5553–5561.

[6] C. C. Romão, W. A. Blättler, J. D. Seixas, G. J. L. Bernardes, *Chem. Soc. Rev.* **2012**, *41*, 3571–3583.

[7] J. S. Ward, R. Morgan, J. M. Lynam, I. J. S. Fairlamb, J. W. B. Moir, *Medchemcomm* **2017**, *8*, 346–352.

[8] C. P. G. Butcher, B. F. G. Johnson, J. S. McIndoe, X. Yang, X.-B. Wang, L.-S. Wang, *J. Chem. Phys.* **2002**, *116*, 6560–6566.

[9] C. P. G. Butcher, P. J. Dyson, B. F. G. Johnson, P. R. R. Langridge-Smith, J. S. McIndoe, C. Whyte, *Rapid Commun. Mass Spectrom.* **2002**, *16*, 1595–1598.

[10] C. S. Hansen, S. J. Blanksby, A. J. Trevitt, *Phys. Chem. Chem. Phys.* **2015**, *17*, 25882–25890.

[11] B. Lucas, M. Barat, J. A. Fayeton, C. Jouvet, P. Çarçabal, G. Grégoire, *Chem. Phys.* **2008**, *347*, 324–330.

[12] D. Imanbaew, J. Lang, M. F. Gelin, S. Kaufhold, M. G. Pfeffer, S. Rau, C. Riehn, *Angew. Chemie Int. Ed.* **2017**, *56*, 5471–5474.
